# Compliance with a three-day course of artesunate-mefloquine combination and baseline anti-malarial treatment in an area of Thailand with highly multidrug resistant falciparum malaria

**DOI:** 10.1186/1475-2875-9-43

**Published:** 2010-02-04

**Authors:** Kanungnit Congpuong, Pongwit Bualombai, Vick Banmairuroi, Kesara Na-Bangchang

**Affiliations:** 1 Bureau of Vector Borne Disease, Department of Disease Control, Ministry of Public Health, Muang District, Nonthaburi, Thailand; 2 Graduate Programme in Biomedical Sciences, Faculty of Allied Health Sciences, Thammasat University (Rangsit Campus), Paholyothin Road, Klong Luang District, Pathumtanee 12121, Thailand

## Abstract

**Background:**

Artemisinin-based combination therapy (ACT) is presently recommended by the World Health Organization as first-line treatment for uncomplicated *Plasmodium falciparum *malaria in several countries, as a mean of prolonging the effectiveness of first-line malaria treatment regimens. A three-day course of artesunate-mefloquine (4 mg/kg body weight once daily for three consecutive days, plus 15 and 10 mg/kg body weight mefloquine on the first and second days) has been adopted by Malaria Control Programme of Thailand as first-line treatment for uncomplicated falciparum malaria all over the country since 2008. The gametocytocydal anti-malarial drug primaquine is administered at the dose of 30 mg (0.6 mg/kg) on the last day. The aim of the present study was to assess patient compliance of this combination regimen when applied to field condition.

**Methods:**

A total of 240 patients (196 males and 44 females) who were attending the malaria clinics in Mae-Sot, Tak Province and presenting with symptomatic acute uncomplicated falciparum malaria, with no reappearance of *Plasmodium vivax *parasitaemia during follow-up were included into the study. The first dose of the treatment was given to the patients under direct supervision. All patients were given the medication for self-treatment at home and were requested to come back for follow-up on day 3 of the initial treatment. Baseline (day 0) and day 3 whole blood mefloquine and plasma primaquine concentrations were determined by high performance liquid chromatography.

**Results:**

Two patients had recrudescence on days 28 and 35. The Kaplan-Meier estimate of the 42-day efficacy rate of this combination regimen was 99.2% (238/240). Based on whole blood mefloquine and plasma primaquine concentrations on day 3 of the initial treatment, compliance with mefloquine and primaquine in this three-day artesunate-mefloquine combination regimen were 96.3% (207/215), and 98.5% (197/200), respectively. Baseline mefloquine and primaquine levels were observed in 24 and 16% of the patients.

**Conclusion:**

The current first-line treatment and a three-day combination regimen of artesunate-mefloquine provides excellent patient compliance with good efficacy and tolerability in the treatment of highly multidrug resistance falciparum malaria. Previous treatment with mefloquine and primaquine were common in this area.

## Background

Malaria chemotherapy is under constant threat from the emergence and spread of multidrug resistance of *Plasmodium falciparum*. Resistance has been observed to almost all currently used anti-malarials. The extensive deployment of anti-malarial drugs in the past fifty years has provided a remarkable selection pressure on malaria parasites to evolve resistance mechanisms. Due to the high resistance level of *P. falciparum *to the most affordable drugs, such as chloroquine and sulphadoxine-pyrimethamine, together with high recrudescence rates with monotherapy with artemisinin derivatives, artemisinin-based combination therapy (ACT) is presently recommended by the World Health Organization as first-line treatment for uncomplicated *P. falciparum *malaria in several countries as a mean of prolonging the effectiveness of first-line malaria treatment regimens [1-4]. Artemisinin derivatives are combined with one of several longer-acting drugs, *i.e*., mefloquine, amodiaquine, sulphadoxine-pyrimethamine, lumefantrine, and piperaquine, which permit elimination of the residual malarial parasites. Among the ACT regimens, the combination of artesunate with mefloquine has remained a highly successful therapeutic regimen in regions habouring the most resistant isolates worldwide particularly in Southeast Asian coutries, such as Thailand and Cambodia [5-9]. It is effective and provides reliably rapid response. The pharmacokinetic advantage of the combination of artesunate with mefloquine is the long half-life of mefloquine (14-21 days), which will eradicate residual parasites remaining from the potent, but short, action of artesunate [10]. In addition, artesunate reduces gametocyte carriage and provides mutual protection for mefloquine against the increased level and spread of resistance [11]. A short course of two or three days is effective and provide reliably rapid therapeutic responses.

In Thailand, where multidrug resistant *P. falciparum *is at high level, the National Malaria Control Programme has adopted artesunate in combination with mefloquine as first-line treatment for uncomplicated falciparum malaria since 1995. The first combination regimen applied initially only in highly mefloquine resistance areas (Tak, Kanchanaburi, Chantaburi, Trat and Mae Hong Son Provinces) was a two-day course of 300 mg (6 mg/kg body weight) artesunate given concurrently on the first day with 750 mg (15 mg/kg body weight) mefloquine, followed by 500 mg (10 mg/kg body weight) mefloquine at six to eight hours apart, then 300 mg artesunate on the second day. In 2004, this combination regimen was adopted to all highly mefloquine resistance areas througout the country, whereas in the low to intermediate resistance areas, the dose of mefloquine was reduced to only a single dose of 750 mg on the first day. Recently in 2008, the combination regimen at the same total dose of 600 mg artesunate and 1,250 mg mefloquine (given as two split doses of 750 and 500 mg), but with a prolonged duration of three days has been implemented all over the country. The gametocytocydal anti-malarial drug primaquine is administered at the dose of 30 mg (0.6 mg/kg) on the last day. It is believed that this regimen will improve the cure rate and delay anti-malarial drug resistance. The concern is however, patient compliance when applied to field condition. The main purpose of the present study was to investigate patient compliance of the current three-day course of artesunate-mefloquine as first-line treatment for uncomplicated falciparum malaria in Thailand.

## Methods

The study was conducted at malaria clinics, Tak Provinces, during March 2008 - February 2009. Malaria is a serious imported medical problem in this area with a peak incidence during May-August and November-January of each year. Most patients are adult males and approximately 55% are caused by *P. falciparum *[12]. The study was approved by the Ethics Committee of the Departement of Disease Control, Ministry of Public Health of Thailand.

A total of 240 patients (196 males and 44 females) presenting with symptomatic acute uncomplicated falciparum malaria (asexual form parasitaemia over 1,000 per microliter blood), who had no history of liver and kidney disease and no previous anti-malarial treatment during the previous four weeks (based on a questionnaire interview), and with no reappearance of *P.vivax *parasitaemia during follow-up were recruited into this study. Median (range) values for age and body weight of the patients were 27(4-69) years and 52(18-65) kg, respectively. The study was conducted under the Monitoring Programme for Antimalarial Drug Resistance of the Ministry of Public Health of Thailand. All of the 240 cases were selected based on their residential areas which would have facilitated the complete follow up. Written informed consents were obtained from all patients before study participation.

Prior to treatment (day 0), blood sample (3 ml) was taken from all patients for determination of mefloquine and primaquine concentrations. Patients were treated with a three-day combination regimen of artesunate and mefloquine. The initial dose of 4 mg/kg body weight artesunate (200 mg, 4 tablets of 50 mg artesunate, Atlantic Pharmaceutical Company, Thailand) and 15 mg/kg mefloquine (750 mg, three tablets of 250 mg mefloquine; Atlantic Pharmaceutical Company, Thailand) were given on the first day (dy 0). Then patients were given artesunate and mefloquine tablets for self-treatment at home. The dose regimen on day 2 was 4 mg/kg body weight artesunate (200 mg, 4 tablets of 50 mg artesunate) and 10 mg/kg body weight mefloquine (500 mg, 2 tablets of 250 mg mefloquine). On day 3, artesunate at the dose of 4 mg/kg body weight was given with 0.6 mg/kg body weight primaquine (2 tablets of 15 mg primaquine; Government Pharmaceutical Organization of Thailand). On day 0, the first dose of the treatment (four tablets of artesunate and three tablets of mefloquine) was given under direct supervision and patient was observed for 30 minutes. If patient vomits within 30 minute, the drug dosages were repeated. When necessary, symptomatic treatment with antipyretic paracetamol, and anti-emetic dimenhydrinate (Dramamine™) was administered.

Patients were requested to return for follow-up in the morning of the third day of treatment (day 3), and on days 7, 14, 21, 28 and 42 or at any time if fever or symptoms suggestive of malaria developed. At each visit, a parasite count was performed on a Giemsa-stained blood film, and a detailed questionnaire for general symptoms was recorded. Blood samples were taken (3 ml) for determination of mefloquine and primaquine concentrations on day 3. Whole blood (1 ml) and plasma (1 ml) samples were stored at -20°C immediately for the analysis of mefloquine and primaquine.

Patients failing to respond to the three-day regimen of artesunate-mefloquine were treated with the second-line treatment for uncomplicated falciparum malaria (quinine plus doxycycline given for seven days). Those who developed *Plasmodium vivax *malaria in their peripheral blood during the follow-up periods were treated with 300 mg (base) of chloroquine to suppress symptoms and a full course of treatment was given at the end of the study period (chloroquine 1,500 mg given over 48 h, followed by 15 mg (base) of primaquine daily for 14 days) and excluded from data analysis.

The clinical outcome of a three-day course of artesunate-mefloquine was evaluated in the group of patients who completed the 42-day follow-up period. The classification of treatment outcomes was based on an assessment of the parasitological and clinical outcome of anti-malarial treatment according to the latest guidelines of WHO. Accordingly, all patients were classified as having an Early Treatment Failure, a Late Clinical Failure, a Late Parasitological Failure, or an Adequate Clinical and Parasitological Response [13]. Patient compliance to treatment medication was defined as his/her reliability in using an exact prescribed medication, together with the levels of the drug taken within the acceptable limits. With the help of a structured questionnaire, patients were interviewed to obtain information on the number of artesunate, mefloquine and primaquine tablets taken. Compliance to the treatment regimen was assessed using Box and Whisker plot by identifying the outlier whole blood mefloquine and plasma primaquine concentrations on day 3 of the initial treatment (1.5-3 box length apart from upper or lower limit line). Mefloquine blood concentrations were measured by high performance liquid chromatography with UV-detection (HPLC-UV) according to the method of Kangwang *et al*, with quantification limit of 3 ng/ml [14]. Primaquine plasma concentrations were measured by high performance liquid chromatography with UV-detection (HPLC-UV) according to the method of Na-Bangchang *et al*, with quantification limit of 1 ng/ml [15].

## Results

All patients had a rapid initial response to treatment with parasites cleared from peripheral blood within 3 days of an initial dose of artesunate and mefloquine. All patients completed the 42 day follow-up period. Two patients had recrudescence on day 28 and 35. The Kaplan-Meier estimate of the 42-day efficacy rate was 99.2% (95% CI 99.0-99.8). No serious adverse event (SAE) was reported during the study. Many adverse events (AE) most likely related to the underlying malaria disease such as headache, muscle pain and anorexia. These symptoms disappeared by day 2 or day 3 after the treatment. Based on questionnaire interview, full compliance of 100% was obtained.

### Mefloquine concentrations

**Figure 1:**
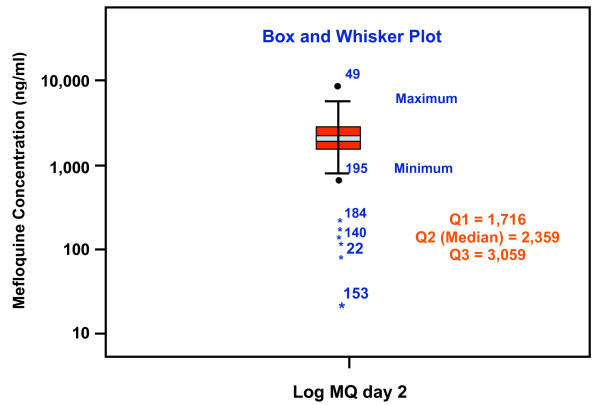
**Box and Whisker plot of whole blood mefloquine concentrations on day 3 after the initial treatment**. Median, 1^st ^and 3^rd ^quatiles = 2,359, 1,716 and 3,059 ng/ml, respectively. Each individual dots represents the case with outlier concentration below or above 1.5-3 box length.

Of the 215 patients whose mefloquine concentrations were measured, 163 (75.8%) had undetectable baseline mefloquine level, whereas 15 (7.0%), 24 (11.6%), and 13 (6.0%) had concentrations of less than 100, between 100 and 500, and more than 500 ng/ml, respectively [median (range):42 (0-2,424) ng/ml]. Since mefloquine is a long half-life drug (14-21 days in patients), the decrease in the concentrations within three days is insignificant and, therefore, day 3 concentrations in patients who had measurable baseline concentrations were estimated by subtraction of baseline concentrations from the measured day 3 concentrations. Figure 1 represents Box and Whisker plot of whole blood mefloquine concentrations on day 3 in 215 cases. The upper, mid and lower lines which represent 1^st^, 2^nd ^and 3^rd ^quatiles were 1,716, 2,359, and 3,059 ng/ml, respectively. Median (range) concentrations on day 3 were 2,359 (27-10,965) ng/ml, with a 95% confidence interval of 1,977-2,353 ng/ml. There were 10 patients with outlier mefloquine concentrations (1.5-3 box length apart from the upper or lower limit line). Two had concentrations above the upper limit (10,000 ng/ml), 7 had concentrations below the lower limit (27, 90, 141, 172, 215, 247, 695 ng/ml), and one had undetectable level of mefloquine (lower than quantification limit). Based on mefloquine concentrations on day 3 with exclusion of pharmacokinetic factor, complaince with mefloquine in this three-day artesunate-mefloquine combination regimen was 96.3% (207/215).

### Primaquine concentrations

**Figure 2:**
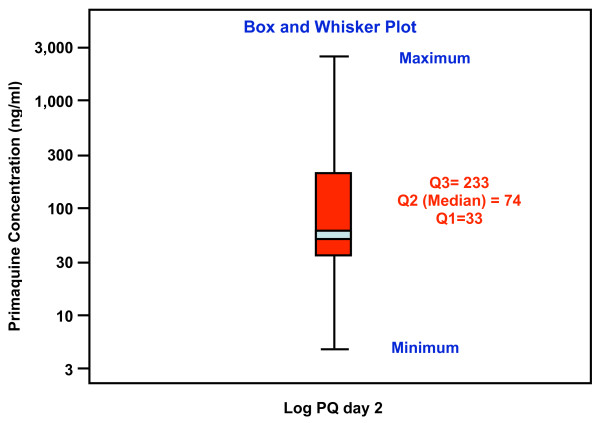
**Box and Whisker plot of plasma primaquine concentrations on day 3 after the initial treatment**. Median, 1^st ^and 3^rd ^quatiles = 33, 74 and 223 ng/ml, respectively. Each individual dots represents the case with outlier concentration below or above 1.5-3 box length.

Of the 214 patients whose primaquine concentrations were measured, 180 (84.1%) had undetectable baseline primaquine level, whereas 29 (13.6%), 4 (1.9%), and 1 (0.5%) had concentrations of less than 100, between 100 and 500, and more than 500 ng/ml, respectively [median (range): 0 (0-1,662) ng/ml]. Figure 2 represents Box and Whisker plot of plasma primaquine concentrations on day 3 in 200 cases. The upper, mid and lower lines which represent 1^st^, 2^nd ^and 3^rd ^quatiles were 1,716, 2,359, and 3,059 ng/ml, respectively. Median (range) concentrations on day 3 were 89 (6-640) ng/ml, with a 95% confidence interval of 75-106 ng/ml. There was no patient with outlier primaquine concentrations, but undetecable concentration was observed in three cases. Whole blood mefloquine concentrations in these cases were as high as 2,669, 2,964 and 6,703 ng/ml. Based on primaquine concentrations on day 3 with exclusion of the pharmacokinetic factor, compliance with primaquine in this three-day artesunate-mefloquine combination regimen was 98.5% (197/200). There was no correlation between the pre-treatment concentrations of mefloquine and primaquine found in this study.

## Discussion

Mefloquine was introduced first as a single-dose therapy for falciparum malaria in Thailand in 1984, but initial high cure rates were not sustained. In a proposition to halt the loss of anti-malarial monotherapies to resistance in rapid succession, the strategy of ACT with mefloquine was developed and was adopted in Thailand in 1994. The current regimen of a three-day combination of artesunate-mefloquine given in total adult dose of 600 mg (12 mg/kg body weight) artesunate and 1,250 mg (25 mg/kg body weight) mefloquine has been implemented by the Bureau of Vector Borne Disease, Department of Disease Control, Ministry of Public Health for the treatment of acute uncomplicated falciaprum malaria since 2008. The main objective was to obtain high cure rate of approaching 100% to prevent the spread of drug resistance. Prior to 2008, the same total dose of artesunate-mefloquine was given but as a shorter course of two days and two split doses of mefloquine (1,250 and 750 mg) given on the same day at 6-8 hours apart instead of two separate days as the current regimen. With the current regimen, in addition to the achievement of high cure rate, the incidence of vomiting due to high dose mefloquine and thus treatment failure is expected to be reduced. Absorption of mefloquine in malaria patients is dose limited and is reduced in the acute phase of illness. Splitting the dose of 25 mg/kg mefloquine improves mefloquine absorption and bioavailability, and thus and the therapeutic response in the treatment of acute falciparum malaria [16-18].

In the present study, a good initial response was observed in all patients where parasitaemia was cleared within three days after the initial dose of artesunate and mefloquine. The present combination regimen of artesunate-mefloquine was shown to improve the cure rate from approximately 87% with the 2 day course to 96.3% in the same study area (Tak province) during the year 2001 to 2002 [7]. There were only two cases with treatment failure (cure rate 99.2%) on day 28, and 35 of treatment. It appears that the cure of this combination is still retained after eight years of its use (Ministry of publc Health, unpublished data). The concern about this three-day course is patient compliance when adopted as a public health policy. The aim of the present study was to assess the patient compliance of the current three-day course of artesunate-mefloquine when applied to field condition. As the combination is not a fixed dose regimen, assessment of blood concentration of only one combination partner, *i.e*. mefloquine with long half-life of 14-21 days [19] may not reflect the real full compliance of the combination regimen. Furthermore, since the half-lives of artesunate and its active plasma metabolite (dihydroartemisinin) are very short (0.5-2 hr), the drug would have been cleared from blood before 24 hours until patients returned for follow-up on the third day of treatment (day 3). Apart from mefloquine, plasma concentrations of primaquine on day 3 was, therefore, also used as a marker of patient's adherence to the three-day regimen with the assumption that if patients took primaquine tablets, it was likely that they would have also taken artesunate tablets on the second and third day. Several methods have been applied for monitoring of compliance to drug treatment particularly in the treatment of chronic diseases. These included clinical judgement of physician, patient self report, clinical response, biochemical measures, pill counts, pharmacy records, electronic medication monitoring devices and measuring of drug concentrations in blood or plasma [20-22]. Most methods except the last one suffer from their subjective evaluation. Measurement of anti-malarial drug concentrations in blood has been applied satisfactorily for monitoring of patient compliance to a 2 day combination regimen of artemether-mefloquine when adopted to field application [23]. In another case, measurement of drug other than the anti-malarial itself, *e.g*. low dose phenobarbital, than the has been applied for monitoring compliance to short course treatment with anti-malarial regimens (5 day course of artesunate and 7 day course of the combination quinine-tetracycline) [24]. The concentrations of artesunate and mefloquine on day 3 (about 24 hours after the last dose of mefloquine and primaquine on day 2) were selected as a time point for monioring the levels of both drugs. Mefloquine is a long half-life drug, therefore, whole blood mefloquine levels on day 3 after the initial treatment could be applied for monitoring of compliance to this combination regimen with good accuracy. Based on mefloquine and primaquine concentrations on day 3 after the intial treatment, patient compliance of as high as 96-98% was achieved when excluding the pharmacokinetic factor due to poor and variable drug absorption in some cases. It is noted however that patients realized that they were participating in a study and may significantly influenced their behaviour (i.e. better compliance). Using the questionnaire interview as a tool to evaluate compliance to the dose regimen, full compliance of 100% was obtained, which means that about 2-4% of non-compliance was undetected by this method. One case with undetectable mefloquine level on day 3 could be definitely classify as non-compliance. The low levels of mefloquine in the seven cases could be due either to non-compliance to the second dose of mefloquine or impaired drug absorption in each individual as none vomited within the first hour. In our previous study, patient compliance with a two-day course of artemether-mefloquine (an initial dose of 300 mg artemether on the first day, followed by 750 and 500 mg meflouine given on the second day at 4-6 hours apart) was investigated in the same area of the country. Median (range) whole blood mefloquine concentrations following the split doses were determined on the third day (1 day after the initial dose of mefloquine) was 2,262 (1,198-3,241) ng/ml. This is considered relatively low when compared with the concentrations observed after three days of an initial dose of mefloquine [median (range) of 2359 (27-10,965) ng/ml]. For primaquine on the other hand, plasma concentration on day 3 may not be absolutely a suitable marker for monitoring of compliance to this combination regimen in this field setting as the half-life of primaquine is relatively short (3.7-9.5 hr) [15]. It has been reported that plasma concentration of primaquine at 25 hr after a single oral dose of primaqune is only approximately 10-15% of the C_max _[15]. The case with undetectable primaquine concentration could be due to pharmacokinetic factors (impaired absorption and/or rapid clearance) and/or genuine non-compliance. Full compliance of approaching 100% with this short course regimen is considered excellent when comparing with compliance to long treatment courses of other regimens, such as a seven-day course of quinine-tetracycine, where prolonged drug administration or a relatively high incidence of cinchonism contributes to about 71.7% compliance in the field trials [25]. This excellent compliance to the treatment regimen may also be due to reduced incidence of adverse effects from mefloquine with the split dose. A simple pre-packaging system and proper counselling could improve compliance with anti-malarial drug treatment [6,26,27]. Co-formulation of the drugs reduces the pill burden and more importantly, eliminates the possibility of patients taking only one component of the combination. In one study, pre-packaging anti-malarial drugs have been shown to improve compliance by approximately 20% in both adults and children [27]. In addition, there were 50% reductions in cost of patients, waiting time at dispensaries and drug wastage at facilities. A new fixed-dose co-formulation of artesunate-mefloquine has been launched by Drugs for Neglected Diseases Initiative (DNDi), which decreases the risk of resistance due to compliance factor [28]. Nevertheless, this strategy may have limitation with regard to practicality of dose adjustment.

It is interesting to note that 24.2 and 15.9% of patients had mefloquine and primaquine levels at baseline pretreatment. Seven% had baseline mefloquine concentrations less than 100 ng/ml, 11.6% had concentrations between 100 and 500 ng/ml, and 13% had concentrations greater than 500 ng/ml. This implies that patients may have received previous treatment with mefloquine longer than 35-48, between 35-48, and within 14 days, respectively [29]. In one of our previous study, it was observed that 10.2, 11.5, 6.0 and 1.1% had baseline whole blood mefloquine concentrations of <100, 100-500, >500-1,000, and >1,000 ng/ml, respectlvely [30]. The lower incidence of observed baseline primaquine level could be explained by the short half-life of primaquine [15]. High incidence of baseline levels in this patient population indicates the high rate of malaria transmission in this area and/or drug resistance. Patients may have received treatment with artesunate-mefloquine treatment with or without primaquine (as a gametocytocide for falciparum malaria or as antirelapse for vivax malaria) from other health services nearby or from illegal anti-malarial drugs available in the markets for the current or previous malaria episode. There has been no data on the adherence to the 14-day course of primaquine for the treatment of vivax malaria, especially primaquine for the treatment of vivax malaria. There was no data on the adherence to the 14 day course of primaquine for the treatment of vivax malaria. Based on the prevalence of primaquine levels on admission of greater than 15%, and when considering the short half-life of this drug, this would suggest that some may have received previous treatment with primaquine within a day before participation in the study. This is of concern as accumulated high level of mefloquine may aggravate high incidence of adverse effects especially the serious neuropsychiatric effect. Based on patients' interview, none received previous treatment with any anti-malarial drug. Measurement of drug levels are, therefore, good confirmation of previous anti-malarial treatment.

## Conclusion

The current first-line treatment three-day combination regimen of artesunate-mefloquine provides excellent patient compliance with good efficacy and tolerability in the treatment of multidrug resistance falciparum malaria in field setting. Previous treatment with mefloquine and primaquine was common in this area. The results obatined are useful for the malaria control programme of Thailand as baseline data for optimization of treatment regimen for uncomplicated falciparum malaria.

## Competing interests

The authors declare that they have no competing interests.

## Authors' contributions

KC participated in the design of the study and performed the statistical analysis. PB conceived of the study, and participated in study coordination. VM carried out the measurement of drug concentrations. KN partcipated in the design of the study, data analysis and drafted the manuscript. All authors read and approved the final manuscript.
